# Changes in the neuropeptide content of *Biomphalaria* ganglia nervous system following *Schistosoma* infection

**DOI:** 10.1186/s13071-017-2218-1

**Published:** 2017-06-02

**Authors:** Tianfang Wang, Min Zhao, Di Liang, Utpal Bose, Satwant Kaur, Donald P. McManus, Scott F. Cummins

**Affiliations:** 1 Genecology Research Centre, Faculty of Science, Health and Education, University of the Sunshine Coast, Maroochydore DC, Queensland 4558 Australia; 2 Institute of Environment, Health and Societies, Brunel University London, Kingston Lane, London, UB8 3PH UK; 3 Molecular Parasitology Laboratory, QIMR Berghofer Medical Research Institute, Brisbane, Queensland 4006 Australia

**Keywords:** *Biomphalaria*, *Schistosoma*, Neuropeptide, Pre-patent infection, Protein-protein interaction, Central nervous system, Proteomics, Host-parasite interaction

## Abstract

**Background:**

Molluscs, including snails, are prone to parasite infection, which can lead to massive physiological and behavioural changes, yet many of the molecular components involved remain unresolved. Central to this point is the neural system that in snails consists of several ganglia that regulate the animals’ physiology and behaviour patterns. The availability of a genomic resource for the freshwater snail *Biomphalaria glabrata* provides a mean towards the high throughput analysis of changes in the central nervous system (CNS) following infection with *Schistosoma* miracidia.

**Results:**

In this study, we performed a proteomic analysis of the *B. glabrata* CNS at pre-patent infection, providing a list of proteins that were further used within a protein-protein interaction (PPI) framework against *S. mansoni* proteins. A hub with most connections for both non-infected and infected *Biomphalaria* includes leucine aminopeptidase 2 (LAP2), which interacts with numerous miracidia proteins that together belong to the immunoglobulin family of cell adhesion related molecules. We additionally reveal the presence of at least 165 neuropeptides derived from the precursors of buccalin, enterin, FMRF, FVRI, pedal peptide 1, 2, 3 and 4, RYamide, RFamide, pleurin and others. Many of these were present at significantly reduced levels in the snail’s CNS post-infection, such as the egg laying hormone, a neuropeptide required to initiate egg laying in gastropod molluscs.

**Conclusions:**

Our analysis demonstrates that LAP2 may be a key component that regulates parasite infection physiology, as well as establishing that parasite-induced reproductive castration may be facilitated by significant reductions in reproduction-associated neuropeptides. This work helps in our understanding of molluscan neuropeptides and further stimulates advances in parasite-host interactions.

**Electronic supplementary material:**

The online version of this article (doi:10.1186/s13071-017-2218-1) contains supplementary material, which is available to authorized users.

## Background

The fresh water snail *Biomphalaria glabrata* is of medical significance as it acts as an intermediate host for the transmission of human intestinal schistosomiasis, caused by *Schistosoma mansoni* [1]. Schistosomiasis remains one of the most intractable public health concerns in 52 endemic countries, at least 258 million people required preventive treatment in 2014 [2]. It is second only to malaria among neglected tropical diseases in its negative impact on global human health [3] and more research is required to enable its control and eventual elimination.

*Schistosoma mansoni* has a complex life-cycle involving two hosts that has been reviewed extensively (e.g. [4]). One critical phase in the life-cycle requires that a miracidium, the waterborne larval stage of *S. mansoni*, locate and infect *B. glabrata* where it can initiate asexual reproduction within the host. Infected snails eventually release free-swimming cercariae that must in turn locate and infect a human host for the cycle to continue.

Sporocyst formation from miracidium within *B. glabrata* alters the snail’s immunity and metabolism, causing parasitic castration such that it can no longer reproduce, yet continues to support the generation of cercariae [5, 6]. Host-defence against invading miracidia appears to rely on a diverse family of fibrinogen-related proteins containing immunoglobulin-like domains [7]. Also, circulating hemocytes present in the snail hemolymph are known to encapsulate miracidia [8]. Such hemocyte-mediated cytotoxicity mechanisms include non-oxidative and oxidative pathways, involving lysosomal enzymes and reactive oxygen/nitrogen intermediates [9, 10]. An investigation of the *S. mansoni* miracidium proteome and its associated pathways, has provided some clues that may help to understand this host-parasite interaction [11].

Neuropeptides encompass a diverse class of chemical messengers that are instrumental in orchestrating complex physiological events from growth to reproduction and immunity. In silico data mining has revealed that at least 41 neuropeptide-like genes are present within *B. glabrata,* encoding precursor proteins that are predicted to release over 300 bioactive cleavage products (Adema et al., under revision). These include the neuropeptides APGWamide, conopressin, elevenin, FMRFamide, gonadotropin-releasing hormone (GnRH), whitnin, NPY and orcokinin. Although neuropeptide transcripts are represented within most of the snail tissues investigated, they are most prominent within the central nervous system (CNS) (23 of 41) and terminal genitalia (21 of 41). The achatin, GnRH, bursicon alpha, GPA2, GPB5, insulin-like peptides 1, 2 and NKY appeared to be exclusive to the snail’s CNS.

In this study, we were interested to know what molecular components were modified in the *B. glabrata* CNS following infection by *S. mansoni* miracidia. To achieve this, peptides were extracted from CNS tissue of non-infected and 12 days infected snails (pre-patent), then analysed by liquid chromatography tandem mass spectrometry (LC-MS/MS), both qualitatively and quantitatively. A protein-protein interaction network was investigated between the snail and parasite to determine putative interaction components. Then, neuropeptide abundance was assessed, with a focus on those implicated in molluscan reproduction. We additionally report the peptidomic characterisation of novel peptides displaying motifs characteristic of neuropeptides.

## Results

### Identification of proteins from the non-infected and infected *B. glabrata* CNS

**Fig. 1:**
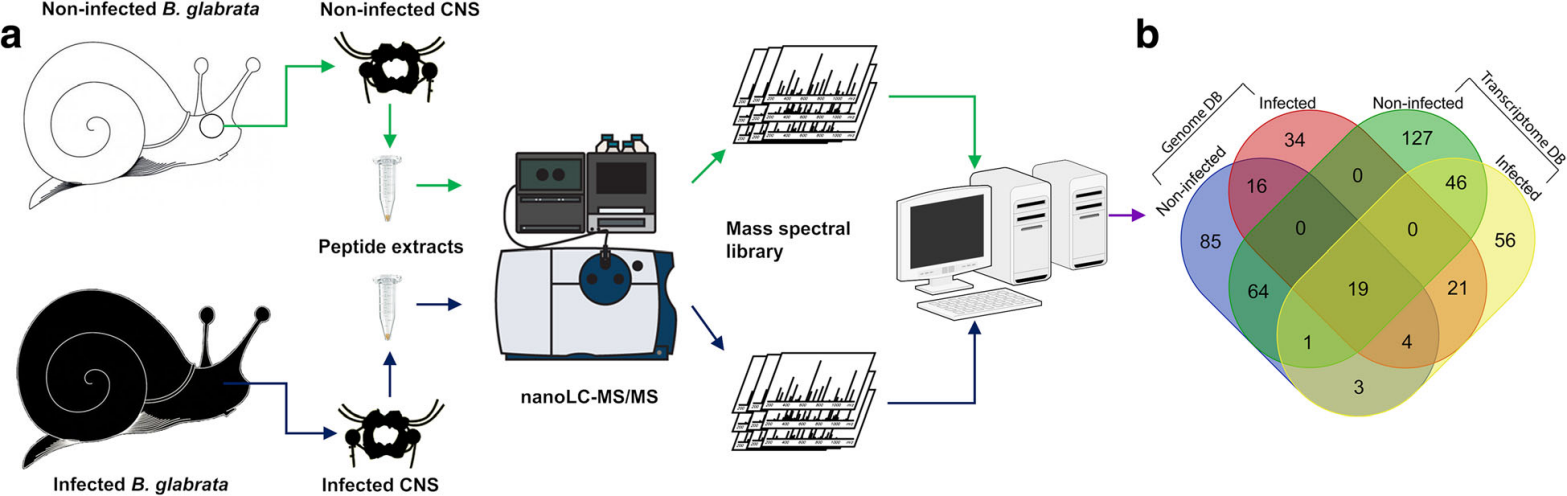
Overall workflow for identification of peptides within non-infected and infected *B. glabrata*. **a** CNS tissues were collected from three batches of snails for each state, respectively; each batch contained 12 individuals. CNS peptides were extracted using acidified methanol and extensively fractionated followed by identification with high-accuracy nano-LC TripleTOF MS. **b** Venn diagram comparison of peptides identified using LC-MS/MS based on genome (version 1.3) and transcriptome-derived protein databases

Biological triplicates of ganglia peptide extracts (non-infected and infected) were extensively fractionated by nano-HPLC, followed by tandem MS as shown in Fig. 1a. All samples were subjected to high-accuracy mass spectrometry, and the raw data were rigorously analysed using PEAKS. Using the genome [12] derived protein database (version 1.3), we identified a total of 125 peptides that matched to 68 precursor proteins (one or more unique peptides with an FDR ≤ 1%) in the non-infected *B. glabrata* CNS. Within the infected pre-patent snail’s CNS we identified 94 peptides from 18 precursor proteins, where only 2 were exclusive to infected CNS. There were 168 sequences supported by 257 peptides identified in non-infected snail’s CNS using the transcriptome-derived protein database, compared to 150 peptides from 69 proteins from infection samples. Fig. 1b shows the results of a comparison between the peptides identified at non-infected and pre-patent infected stages using two databases (for more details of peptide/protein identification, see Additional file 1: Table S1). The peptides and the corresponding precursor proteins only had matches in transcriptome derived protein database.

### Protein-protein interaction (PPI) of identified *B. glabrata* CNS proteins with *S. mansoni* miracidium or sporocyst proteins

**Fig. 2:**
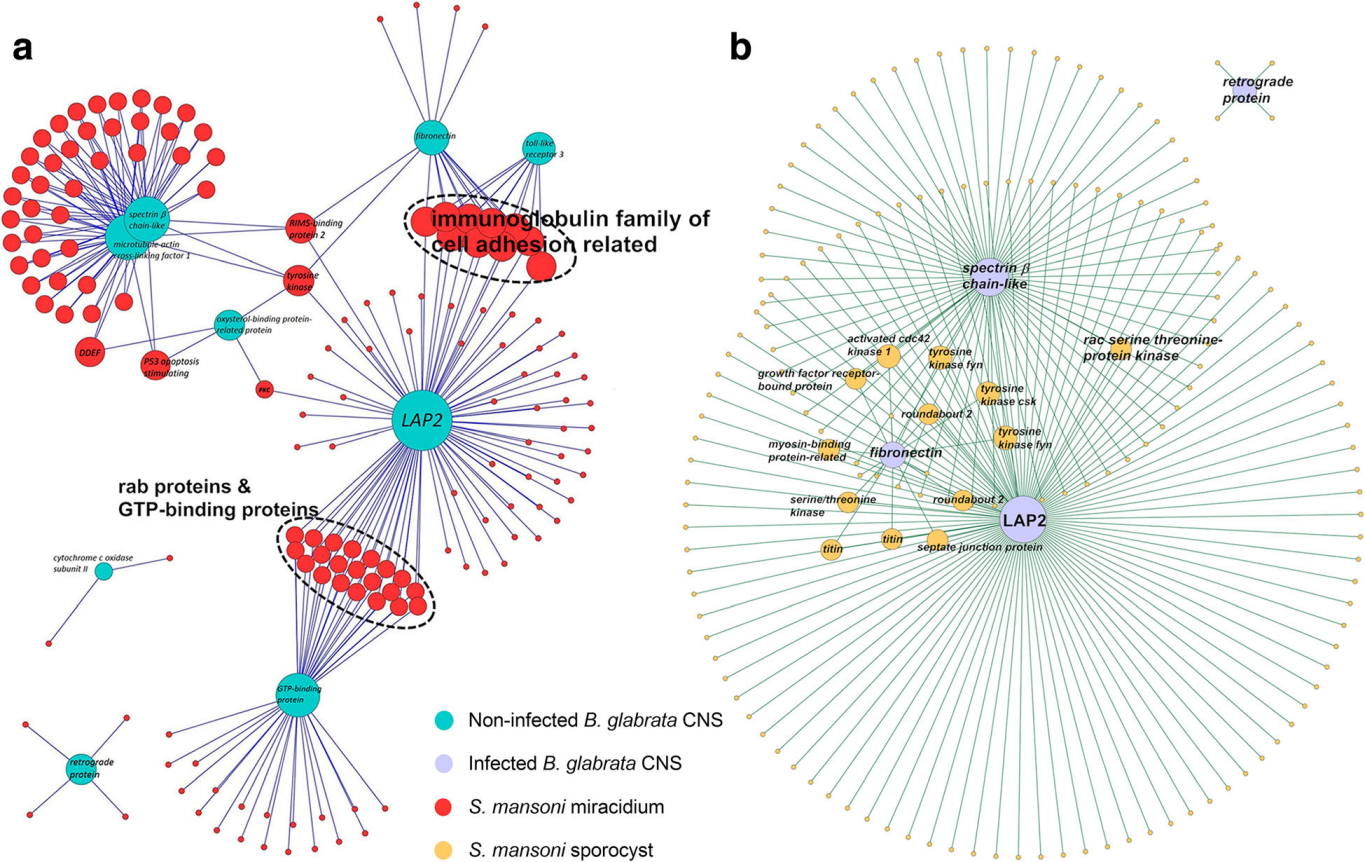
Protein-protein interactions of (**a**) *S. mansoni* miracidium proteins with non-infected *B. glabrata* CNS proteins and (**b**) *S. mansoni* sporocyst proteins with infected CNS proteins identified. The domain-domain interaction and gene ontology annotations using DOMINE database (download on July 20th, 2014) and HMMER based on the Pfam database (version 37) were used, respectively. Each PPI with 6 or more domain-domain interaction was chosen to display, allowing for a more accurate prediction. Topological analyses were performed to explore the potential function in our constructed network using the NetworkAnalyzer plugin in Cytoscape 2.8.2

PPIs using proteins identified from the non-infected snail’s CNS with *S. mansoni* miracidium proteins [11] are shown in Fig. 2a. Two hubs in the PPI map from the snail cluster together, representing cytoskeletal protein spectrin and microtube actin cross-linking factor 1 (MACF1). For the PPIs of infected snail’s CNS and *S. mansoni* sporocyst, no MACF1 is present (Fig. 2b). One of the hubs with most connections for both non-infected and infected CNS is the leucine aminopeptidase 2 like protein (LAP2). The PPIs of the entire *S. mansoni* proteome *versus* those *B. glabrata* CNS proteins identified are shown in Additional file 2: Figure S1 (for protein accession names see Additional file 1: Table S1 and *S. mansoni* protein annotations see Additional file 3: Table S2).

Those *S. mansoni* miracidium proteins that may interact with both *B. glabrata* LAP2 and GTP-binding proteins consist of rab and GTP-binding protein cluster. The proteins that may interact between LAP2 and fibronectin/toll-like receptor belong to a cluster of immunoglobulin family of cell adhesion related proteins, including *S. mansoni* hemicentin-2, roundabout 2, brother of cdo, netrin receptor dcc, titin, nephrin, receptor-type tyrosine-protein phosphatase and twitchin, while the PPIs of LAP2 and fibronectin were also detected in the infected snail’s CNS with *S. mansoni* sporocyst (Fig. 2b), with the *S. mansoni* septate junction protein additionally involved. The three hubs connecting the two major functional modules in Fig. 2a were rims binding protein 2 and tyrosine kinase (*S. mansoni*), and oxysterol-binding protein-related protein 9 (*B. glabrata*), which was missing from the PPI of infected snail’s CNS. Cytochrome *c* oxidase, acting as a transmembrane proton pump building an electrochemical gradient using chemical energy from the reduction of O_2_ [13], is another node not present (Fig. 2b). There are a total of 60 interactions that can be observed between *Bg-*spectrin and sporocyst proteins.

### Neuropeptides identified in *B. glabrata* CNS by MS/MS

**Fig. 3:**
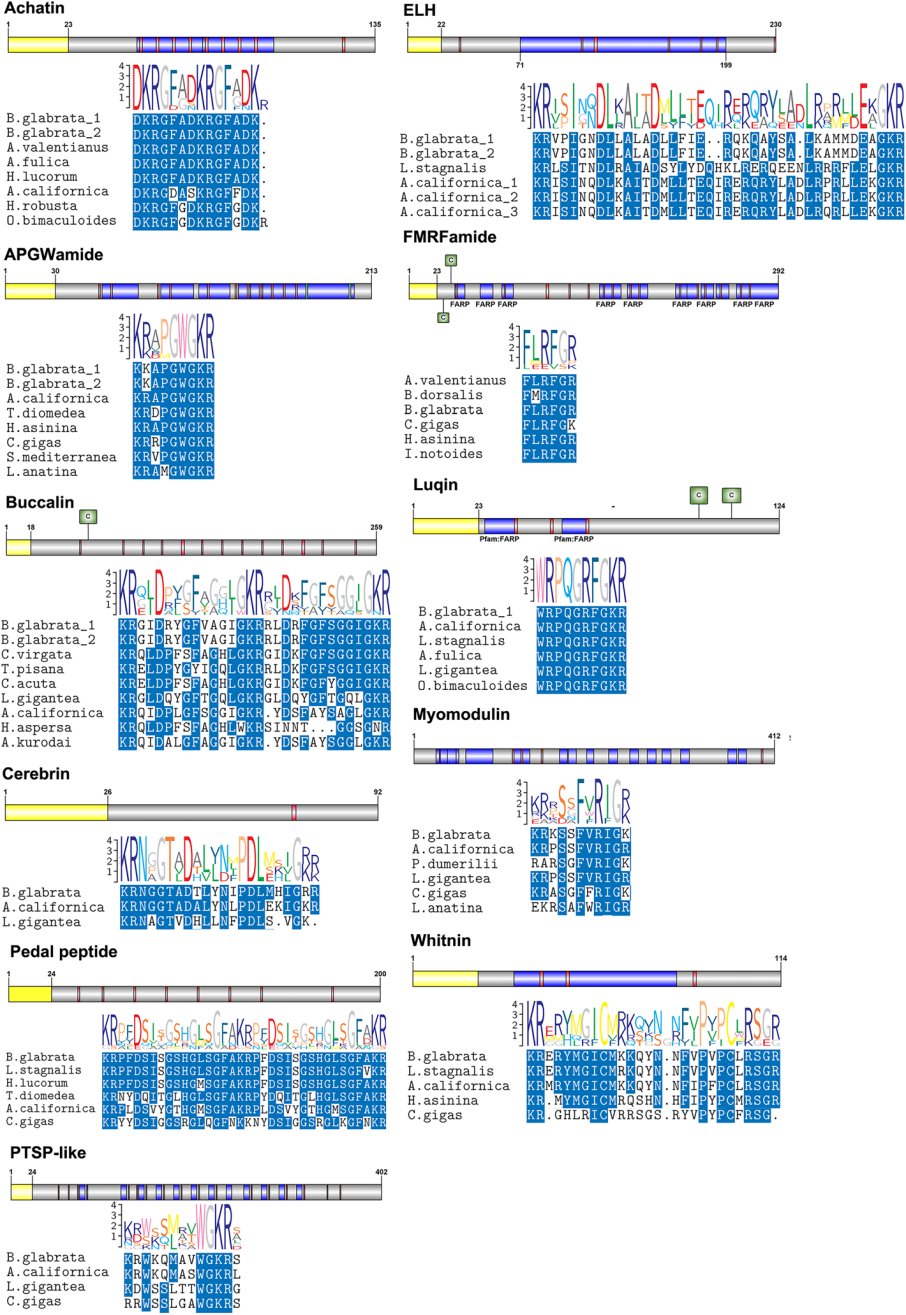
Schematic representation of *B. glabrata* neuropeptide precursors and multiple sequence alignment of bioactive peptide region with other molluscs. For each neuropeptide, a schematic precursor protein and multiple sequence alignment with other molluscs shown. Signal peptide (*yellow*); bioactive peptides (*blue*); cleavage site (*vertical line*); C, represents cysteine residues. In multiple sequence alignment, blue shading provides amino acid conservation. Sequence logo above alignments also show areas of high conservation

Numerous *B. glabrata* peptides identified through MS/MS did match with molluscan neuropeptide precursors. These are not represented within the PPI network since binding partners, if any, are currently unknown. Neuropeptide precursors from the *B. glabrata* genome were annotated with signal sequences and cleavage sites by SignalP and Neuropred, and those detected were listed in Additional file 4. Of these neuropeptide genes, our LC-MS/MS study identified 34 within non-infected snail’s CNS, and 31 within the infected snail’s CNS (Table 1). Neuropeptides that have been implicated in molluscan growth or reproduction were analysed by comparative sequence analysis and showed a precursor organisation that is consistent with homolog neuropeptides and strong amino acid conservation of the bioactive peptide regions (Fig. 3).

**Table 1:** Summary of *Biomphalaria glabrata* neuropeptide precursors annotated from the genome, and those identified by LC-MS/MS in current study indicated by “+”; “–"indicates not detected

Name	CNS non-infected	CNS infected	Name	CNS non-infected	CNS infected
AAP12	+	+	Insulin-like peptide 1	+	−
Achatin	+	+	Insulin-like peptide 2	−	−
Adipokinetic Hormone	−	−	Insulin-like peptide 3	+	−
Allatotropin-like	−	+	Insulin-like peptide 4	−	−
APGWamide	+	+	LFRFamide	+	+
Buccalin 1	+	+	Luqin	+	+
Buccalin-like	+	+	Myomodulin 1 (FVRI)	+	+
Bursicon alpha	−	−	Myomodulin 2	+	+
Bursicon beta	−	−	Myomodulin 3	−	−
CCAP	+	+	NdWF	+	+
Cerebrin	+	+	NKY	−	+
Conopressin	−	−	NPY	+	−
Elevenin	−	−	Pedal peptide 1	+	+
ELH 1	−	−	Pedal peptide 2	+	+
ELH 2	+	+	Pedal peptide 3	+	+
Enterin	+	+	Pedal peptide 4	+	+
FCAP 1	+	+	PKYMDT	+	−
FCAP 2	+	+	Pleurin	+	+
FMRFamide	+	+	Prohormone-1	+	+
Fulicin-like precursor (LRNFVamide)	+	+	PTSP-like	+	−
FFamide	−	+	PRQFVamide	+	−
FVRIamide	+	+	sCAP_1	+	+
GGNG	−	−	sCAP_2	+	+
GnRH	−	−	Schistosomin	−	−
GPA2	−	−	Whitnin /SPTR /Proctolin	+	+
GPB5	−	−	Summary total	34	31

**Fig. 4:**
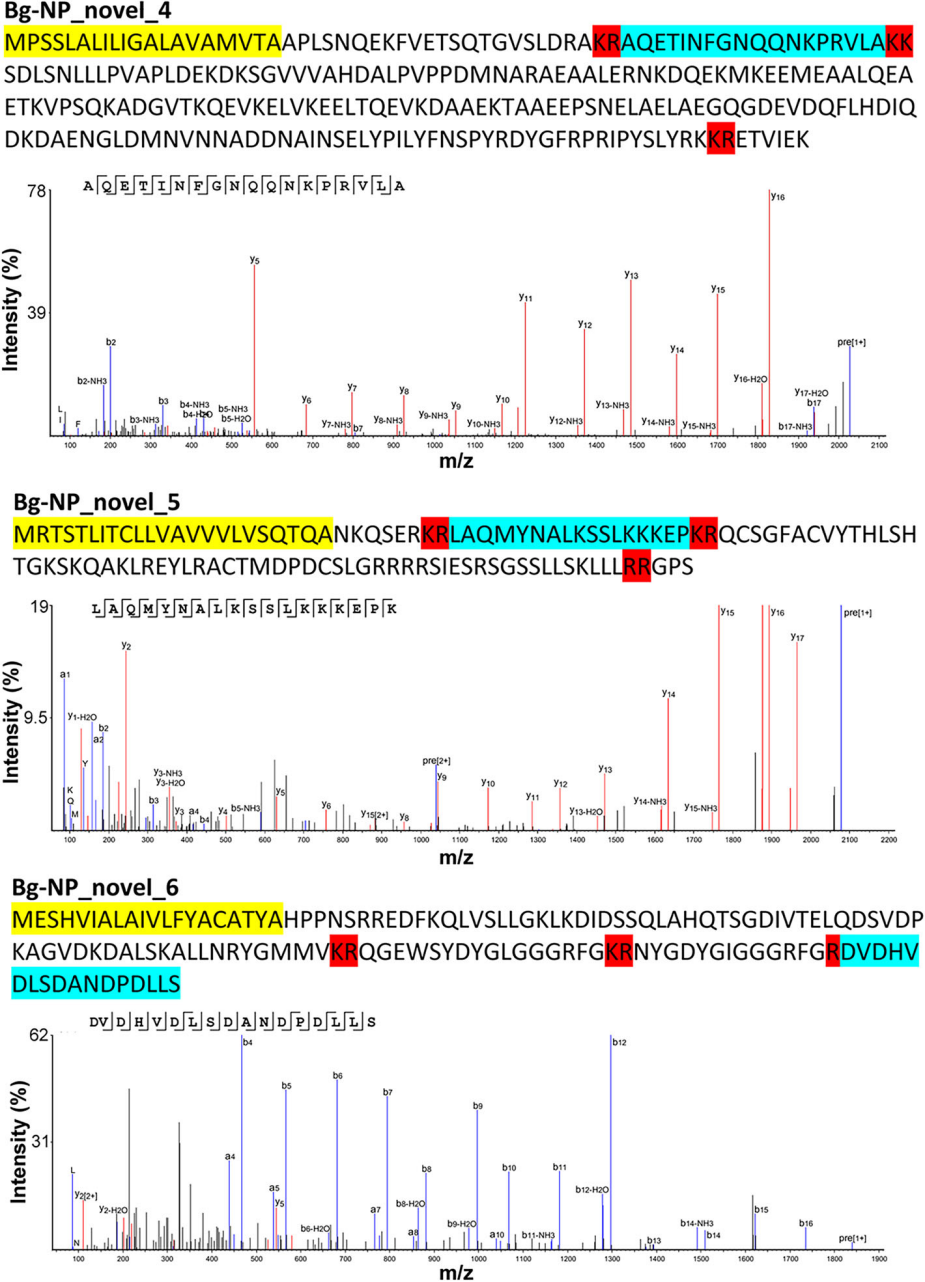
Sequences and MS/MS fragmentation spectra of three novel neuropeptides in *B. glabrata* CNS as determined by ESI-TripleTOF: Bg-NP_novel_4 - AQETINFGNQQNKPRVLA *m/z* 676.70, y2-y16 was detected, relatively abundant compared to the incomplete b-ion series b2-b5; Bg-NP_novel_5 - LAQMYNALKSSLKKKEPK *m/z* 520.06, all y-ions, b2-b4 ions were detected; and Bg-NP_novel_6 - DVDHVDLSDANDPDLLS *m/z* 920.40, b2, b4-b16, y13 and y16 were detected. Signal sequences (*yellow*), cleavage sites (*red*), and bioactive peptide (*blue*) supported by ESI-TripleTof MS/MS analysis

In addition to those known molluscan neuropeptides, several CNS peptides were identified that are likely processed from precursors with characteristics similar to neuropeptide precursors (i.e. signal peptide and peptide cleavage sites). The accessions of these proteins are shown in Additional file 5: Table S3 (named as Bg-NP_novel_1 to 9), supported by 23 peptides identified by LC-MS/MS. The primary sequences and MS/MS spectrum of three novel neuropeptides identified are shown in Fig. 4. Eight of the full-length sequences of these proteins were derived from the *B. glabrata* transcriptome (Vectorbase) and two from the genome annotation. All of these proteins were found in the non-infected CNS, and seven proteins were identified from the infected snail’s CNS, except Bg-NP_novel_7, 9 and 10 (Additional file 5: Table S3). All bioactive peptides that are predicted to be cleaved from these proteins (NeuroPred) are listed in Additional file 5: Table S3.

### Differential abundance of neuropeptides in non-infected and pre-patent infected *B. glabrata* CNS

**Fig. 5:**
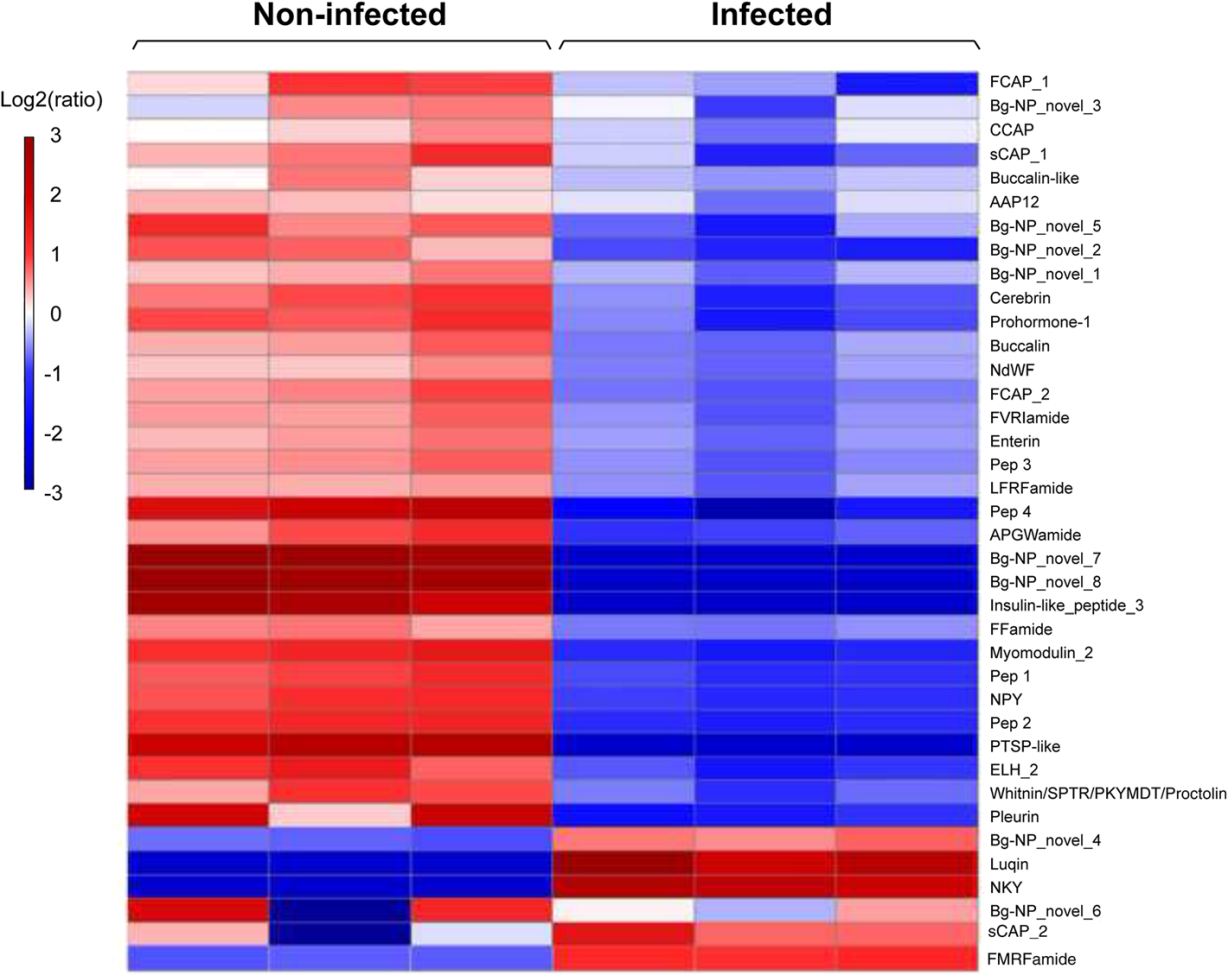
Neuropeptide precursor abundance in the *B. glabrata* CNS of non-infected *versus* at pre-patent stage, based on LC-MS/MS quantitative analysis. The label-free quantification module (PEAKS Q) of PEAKS v7.0 was used to compare relative concentrations of proteins at the two stages. Biological triplicates of each stage were used in tandem repeats and compared. Proteins with fold change ≥1.5 were shown (see Additional file 1: Table S1 for more details)

Potential neuropeptide precursors identified within the *B. glabrata* CNS were quantified using a label-free method based on the relative intensities of peptide features detected in triplicate samples identified by MS/MS. After data quality and fold change (≥ 1.5) filtration, the relative abundance of the neuropeptide precursors within infected and non-infected CNS was compared in Fig. 5. Amongst the neuropeptide precursors that were identified as differentially expressed in the *B. glabrata* CNS were egg laying hormone (ELH_2), whitnin (SPTR/PKYMDT/proctolin), myomodulin 2, FMRFamide, APGWamide, pedal peptide 1 (Pep 1), enterin, cerebrin, buccalin, insulin-like peptide 3, Pep 2, PTSP-like, Pep 3, Pep 4, NKY, luqin (abdominal ganglion neuropeptides L5–67), NPY, prohormone-1, AAP12, NdWF, pleurin, CCAP, sCAP 1 and 2, FVRIamide and LFRFamide. These have been identified and reviewed in several molluscan investigations [14–20]. The majority of the neuropeptides in the CNS were significantly less abundant at 12 days post-infection, such as Pep 1, Pep 2, NPY, PTSP-like, Pep 4, AAP12 and pleurin. The neuropeptide precursors that had slightly decreased included whitnin, Pep 3, NdWF, enterin, buccalin-like and CCAP. A few neuropeptides showed a relative increase in infected snail samples, including FMRFamide, luqin, NKY and sCAP 2. In addition, Bg-NP_novel_1, 2, 3, 5, 7 and 8 showed higher abundance in non-infected CNS, while there was more Bg-NP_novel_4 identified from infected CNS. Bg-NP_novel 6 was variable across biological replicates.

Additional file 6: Table S4 separates these neuropeptides by their precursor and includes those identified by LC-MS/MS. Several neuropeptides were mutually identified in both non-infected and infected snail’s CNS such as 8 buccalins, 2 ELH peptides, 8 enterins and 3 pleurins. Neuropeptides identified in non-infected CNS only, included 2 cerebrins [NGGTADTLYNIPDLMHIamide and NGGTADTLYNIPDLM(+15.99)HIa, where (+15.99) represents oxidation of methionine], ELH and its truncated form [VPIGNDLLALADLLFIERQKQAYSALKAM(+15.99)MDEAa and FIERQKQAYSALKAM(+15.99)MDEAa, respectively], 4 myomodulin peptides [PMNM(+15.99)LRLa, PMNMLRLa, SMKM(+15.99)LRLa and PM(+15.99)NMLRLa]. NPY was exclusively identified in non-infected CNS, while NKY supported by three MS peptides (GDKDDLYSAILQAAESPS, TYATTDATLETILNVLKSHAQSLRQLESTVYEQ and TYATTDATLETILNVLKSHAQSLRQLESTVYEQa) was only detected at pre-patent infection stage.

## Discussion

The CNS of snails consists of several ganglia that control metabolic activities within the foot, mantle, pallial cavity, and visceral organs [21]. In this study, we provide a comprehensive analysis of the *B. glabrata* CNS peptides, including those that may interact with *S. mansoni* miracidia upon infection, as well as the neuropeptides that vary in abundance post-infection.

PPI analysis can provide valuable insight into the molecular physiological mechanisms of infection. Our identification of *B. glabrata* CNS proteins allowed for a semi-targeted PPI analysis with those proteins known to be present in *S. mansoni*. Our PPI network analysis identified the node *Bg*-LAP2 as the protein with highest neighbour connectivity. LAP2, like other aminopeptidases, serves vital roles in processing hormones, neurotransmission and immunological regulation, by catalysing the hydrolysis of N-terminal amino acid residues of proteins and peptides [22]. Although not characterised as LAP2, *Biomphalaria* seemed to have increased hemolymph levels of aminopeptidase following infection by bacteria [23] or *Echinostoma lindoense* [24]. In another gastropod mollusc, *Aplysia*, hemolymph LAP-like protein appears to degrade biologically active alpha-bag cell peptide fragments that along with the ELH peptide, regulate egg laying behaviour [25]*.* It has also been found that LAP2 has highest abundance in the digestive gland of mussel *Mytilus edulis* and its activity positively correlates with rate of whole-body protein turnover, being one of the four main lysosomal proteases associated with 73% of maintenance energy expenditure, which suggests its vital role in metabolic process [26].

Those *S. mansoni* miracidium proteins that cluster and interact with *Bg-*LAP2, fibronectin and toll-like receptor 3 include the *Sm-*netrin receptor, twitchin, roundabout 2, titin, nephrin and hemicentin-2-like proteins (see Fig. 2a). The interactions involving *Bg-*fibronectin, *Sm*-roundabout 2 and titin are sustained into the infection stage with sporocyst. Furthermore, there are significantly more sporocyst proteins (94) that appear to be exclusively interacting with *Biomphalaria* LAP2 than miracidium proteins (49), including various kinases, as well as a few receptors. Thus, it is possible that sporocysts interfere with *Bg-*LAP2 more intensely, thus influence the snail’s physiological activities of regulating neuropeptides for development and reproduction, or metabolism; as a result, there is likely impact on energy production pathways.

We found two hubs in the PPI map from the snail that cluster together, representing the cytoskeletal protein spectrin and MACF1. The role of MACF1 in molluscs remains unclear, although it is known to interact with actin and microtubule cytoskeletons in mammals [27, 28], where it is involved in the translocation and subsequent binding of the Axin complex to LRP6 at the cell membrane [29]. Specifically, MACF1 associates with glycogen synthase kinase 3 [30], and plays critical roles in neuronal migration in the developing brain through its interaction with the Wnt signalling pathway and control over microtubule stability [29]. It is also known that the Band 4.1-domain-containing protein (Bili) acts as a negative regulator of Wnt/β-catenin signaling due to the recruitment of Axin to LRP6 [31]. Thus, we may propose that the absence of MACF1 post-infection suggests possible inhibition of the Wnt signalling pathway, as shown in Additional file 7: Figure S2, where LRP6 phosphorylation might be blocked.

A similar scenario occurs for the oxysterol-binding protein-related protein 9 (ORP9), which was not detected post-infection. Again, its function has not been described in molluscs, yet in mice it mediates transport of sterols between the endoplasmic reticulum and *trans*-Golgi/TGN [32]. The absence of ORP9 within the infected snail’s CNS suggests that the transport of sterols could be suppressed. We found that ORP9 interacts with *S. mansoni* DDEF (development and differentiation-enhancing factor), P53 apoptosis stimulating protein, tyrosine kinase and protein kinase C (PKC). Apoptosis is known to be an important biological process that aids survival and development of *Schistosoma* [33], and the apoptosis-stimulating protein P53 can specifically enhance p53-induced apoptosis [34]. *Sm-*PKC relates directly to movement and reproduction of *S. mansoni* [35], while *Sm-*tyrosine kinases play key roles in reproduction [36].

Neuropeptides control numerous physiological activities and therefore we targeted these for quantitative analysis. Our study clearly shows that a significant modification occurs in the abundance of most *B. glabrata* neuropeptides at pre-patent infection stage. Several of these neuropeptides have been implicated in molluscan reproductive processes, which accords with the report that infection inhibits *B. glabrata* reproduction through suppression of gonad maturation [37]. One neuropeptide of significance is ELH, a well-known inducer of egg laying in the aquatic gastropod snails *Aplysia* and *Lymnaea* (called CDCH in *Lymnaea*, caudodorsal cell hormone) [38–40], and the gene has also been identified in the bivalve molluscs *Crassostrea gigas* and *Pinctada fucata* [18]. The function of *Bg*-ELH is still unknown, although it has enough sequence similarity compared with other gastropod ELH to suggest it may also facilitate egg laying in *Biomphalaria*. Thus, its down-regulation within the infected snail’s CNS would impact reproduction, which is in accordance with a recent study reporting significant impairment of *B. glabrata* egg laying at around seven days post-exposure to miracidia, including a decreased (*P* < 0.05) number of eggs per egg mass and number of eggs per snail within the first two weeks [41].

The neuropeptide, APGWa has also been implicated in reproduction. It was first discovered from the ganglia of the gastropod *Fusinus ferrugineus* [42] and later in other gastropods, including *Lottia*, *Aplysia, Lymnaea* and *Haliotis* [20, 43–45] and the bivalve *C. gigas* [18, 46]. APGWa is known to activate genital eversion in *Lymnaea* [47], spermiation in *Helix aspersa,* spawning in male *Haliotis* [48], and regulates egg transport and spawning in female *C. gigas* [46]. Whilst the oyster APGWa preprohormone contains tetrapeptides that deviate from the APGWa (i.e. KPGWa, RPGWa, SPGWa), the *Bg-*APGWa preprohormone contains only APGWa, a feature consistent with other gastropods [20, 44, 49]*.*

Our analysis identified extensive peptide matches to neuropeptide families Pep 1, 2, 3 and 4 (10 to 14-amino acid peptides with a high degree of sequence similarity in each family), and similar peptides have previously been described for *Aplysia* [50] and *Helix* [51]. Peps were originally isolated from neurons ‘Pd 5’ and ‘Pd 6’, located in the pedal ganglia of the gastropod *Tritonia diomedea* [52], which are active during locomotion [53]. They are also found them to innovate the ciliated foot [54] where they accelerate the beat rate of ciliated foot epithelium cells [55, 56] and the epithelium of the salivary duct [57]. Significant sequence homology is observed at the C-termini of the Pep 3 sequences of gastropod species with several amino acid residues being completely conserved. The extensive sequence homology amongst the Pep 3 supports the hypothesis that they serve an important biological role and that this role is conserved.

Other neuropeptides we identified as being decreased 12 days post-infection have been associated with animal growth, including cerebrin and myomodulin in other gastropod snails. In *Aplysia*, cerebrin can induce arousal behaviours, similar to those obtained when offered food [58]. Moreover, *Aplysia* myomodulin 2 has multiple functions in behavioral plasticity, and its high expression in pedal-buccal projection neurons suggests it acts as a novel source of extrinsic modulation of the feeding system [59]. The significant down-regulation of these two neuropeptides indicates that the feeding activity of *B. glabrata* post-infection may be impaired. In addition, FCAP-1 and 2 were found to be less abundant in infected snails (Fig. 5). In *Aplysia*, FCAPs contribute to the induction and maintenance of food-induced arousal [19]. If FCAP-1, 2 plays a similar role in the *B. glabrata*, this would further suggest that the arousal behaviours induced by feeding in infected snails were inhibited at pre-patent stage [41].

Neuropeptides including NdWFa, AAP12 and pleurin were found to down-regulate in our study; they are known to regulate various physiological aspects in other molluscs, such as cardioexcitation [60] and participating in the calcium regulatory pathways or calcium homeostasis in terrestrial snails [61]. AAP12 has recently been associated with aestivation in the land snail *Theba pisana* [14]. We suggest that low-no production of these neuropeptides in *Biomphalaria* could have widespread implications in the snail’s functioning and biology.

Luqin, FMRFa and NKY precursors were increased at pre-patent infection stage. Luqin has been suggested to enhance tetanic contraction, phasic contraction of the *Mytilus* anterior byssus retractor muscle produced by repetitive electrical stimulation [62]. The tetrapeptide FMRFa is known to evoke contraction of the smooth muscles in different molluscs [63, 64]. FMRFa has also been shown to increase in the hemolymph of metabolically active *Helix aspersa*, leading to a decrease in glucose [65]. Thus, the increment of FMRFa-related peptides might suggest that the infected snails may become metabolically more active during this period of parasite infection.

Unlike previously reports of Pep 3, most of the novel Pep 3 identified in this study share a conserved N-terminal region of RFDRI (except RFDSISDSSAFNHFa). Conserved amino-terminal Arg-containing Pep 3 is caused by a change of a single codon in Pep 3. As shown in Additional file 6: Table S4, thirteen Pep 3 were observed, including 7 more commonly found N-terminal Pro-containing Pep 3 such as PFDRIGTSSFTSFa (*m/z* 730.84) and PFDRIGSSAFTSFa (*m/z* 715.84), and N-terminal Arg-containing Pep 3, such as RFDRIDRGSAFSRFa (*m/z* 432.97), RFDSISDSSAFNHFa (*m/z* 543.57) and RFDRISKNSQFNPFa (*m/z* 585.62). In addition, there was one de novo sequenced peptide RFDSISDSSAFNRFa (*m/z* 549.94) present in non-infected snail’s CNS also possessing the characteristics of Pep 3, with N-terminal Arg. The detection of these novel peptides provided validation of N-terminal Arg-containing sequences in Pep 3 detected in the CNS of *B. glabrata.*

## Conclusions

We have performed an investigation of *B. glabrata* CNS peptides and proteins, and their association with *S. mansoni* infection by comparative LC-MS/MS analysis, both qualitatively and quantitatively. A PPI analysis has revealed those proteins are relevant to infection. It appears that the majority of neuropeptides analysed were significantly down-regulated post-infection, suggesting an infection-induced physiological variation in the *B. glabrata* CNS at pre-patent stage. We also describe the identification of a Pep 3 containing an N-terminal Arg. Our study not only significantly expands the catalogue of neuropeptides present in *B. glabrata*, but also provides a foundation to clarify precisely what the extracellular CNS peptides and precursor proteins are, and what roles they may play in this snail.

## Methods

### Animal rearing and tissue collection

The Puerto Rican strain of *S. mansoni* is maintained in ARC Swiss mice and *B. glabrata* snails at QIMR-Berghofer Medical Research Institute (QIMR-B) from stock originating from the National Institute of Allergy and Infectious Diseases Schistosomiasis Resource Centre, Biomedical Research Institute (Rockville, Maryland, USA). *B. glabrata* (strain BB02) were housed at QIMRB during October 2015, and maintained in flow-through aquarium tanks in a constant temperature room set to 25 °C, and fed to satiety on lettuce. For collection of CNS of ‘non-infected’ snails, animals were removed from aquaria, and were quickly killed upon removal of CNS, which was then snap frozen in liquid nitrogen. For collection of CNS of ‘infected’ snails, animals were infected with *S. mansoni* miracidia using standard protocols [66], the CNS was removed at 12 days post-infection and then snap frozen in liquid nitrogen. Infection was assessed by compression of tissues between two glass plates and examination under a stereoscopic microscope to confirm the presence of sporocysts. In total, CNS was collected from three batches of non-infected and infected snails, where each batch contained 12 snails (at day zero, shell diameters of 8–10 mm, and at day 12, shell diameters of 10–15 mm).

### Peptide isolation and LC-MS/MS analysis of normal and infected *B. glabrata* CNS

The overall experimental procedure to identify and quantify CNS neuropeptides encoded within the *B. glabrata* genome is outlined in Fig. 1. Frozen samples of CNS were ground to a powder under liquid nitrogen in a mortar, transferred into a microfuge tube then quickly weighed in a precooled beaker. They were homogenized thoroughly in extraction buffer (90% methanol, 9% glacial acetic acid in MilliQ water) in a 1:5 w:v ratio. Crude extracts were then sonicated with three pulses, 30 s each, and centrifuged for 20 min (16,000×*g*, 4 °C). Supernatant was collected and lyophilised.

CNS extracts were analysed by LC-MS/MS on a Shimadzu Prominance Nano HPLC (Nakagyo-ku, Japan) coupled to a Triple Tof 5600 mass spectrometer (AB SCIEX, Concord, Canada) equipped with a nano electrospray ion source. The protocol has been detailed elsewhere [67]. Briefly, approximately 6 μl of each extract was injected and de-salted on the trap column using solvent A [0.1% formic acid (aq)] before entering a nano HPLC column (Agilent Technologies, Mulgrave, Australia) for mass spectrometry analysis. Peptide elution used a linear gradient of 1–80% solvent B [90:10 acetonitrile: 0.1% formic acid (aq)] over 120 min at 300 nl/min flow rate. Solvent B was then held at 80% for 5 min to wash the column and then returned to 1% solvent B for equilibration prior to the next sample injection. The mass spectrometer acquired 500 ms full scan TOF-MS data followed by 20 by 50 ms full scan product ion data. Full scan TOFMS data were obtained over the mass range 350–1800 and for product ion MS/MS 100–1800. Ions observed in the TOF-MS scan exceeding a threshold of 100 counts and a charge state of +2 to +5 were set to trigger the acquisition of product ion. The data were acquired and processed using Analyst TF 1.5.1 software (AB SCIEX, Concord, Canada).

Fragmentation data were analysed by PEAKS v7.0 software (BSI, Waterloo, ON, Canada). Sequences of peptides were determined by comparing the fragmentation patterns with those of proteins annotated from the *B. glabrata* genome (version 1.3, 14,223 entries), and also sequences derived from transcriptomes (containing 432,561 entries) within twelve tissues (https://www.vectorbase.org/). Search parameters were as follows: no enzyme was used; variable modifications included methionine oxidation, conversion of glutamine/glutamate to pyroglutamic acid, deamidation of asparagine and peptide amidation. Precursor mass error tolerance was set to 0.1 Da and a fragment ion mass error tolerance was set to 0.1 Da. De novo sequencing, database search and characterising unspecific post-translational modifications (PTMs) were used to maximise the identifications; false discovery rate (FDR) was set to ≤1%, and the individual peptide ion score [-10*Log(*p*)] was calculated accordingly, where *p* is the probability that the observed match is a random event. Proteins and their supporting peptides were obtained and analysed.

### Protein-protein interaction (PPI) network

To present a PPI map, we extracted PPIs of *B. glabrata* proteins identified (non-infected/infected) *versus* *S. mansoni* proteins, with the miracidium proteins [11] investigated particularly, using the domain-domain interaction and gene ontology annotations. To this aim, we first adopted HMMER [68] to annotate all the known protein domains based on the Pfam database (Version 37) [69]. Using the annotated protein domain information, we used the high confidence domain-domain interaction from the DOMINE database (download on July 20th, 2014) [70] to connect those proteins with domain-domain interaction relationship. Since a protein often contains multiple domains, we required each PPI with 6 or more domain-domain interaction support to allow for a more accurate prediction. The protein database of *S. mansoni* was downloaded (from http://metazoa.ensembl.org/, ASM23792v2.27), from which the miracidium proteins were identified [11] and used in this study. The sporocyst protein database with full-length protein sequences was derived from its transcriptome [71], by searching the ORFs against ASM23792v2.27 using BLAST with eValue ≤10^-10^. In addition, topological analyses were performed to explore the potential function in our constructed network using the NetworkAnalyzer plugin in Cytoscape 2.8.2 [72]. The final network visualisation was performed using Cytoscape.

### Quantitative peptide analysis

The quantitative analysis of proteins was carried out using the label-free quantification module PEAKS Q [73] of PEAKS Studio v7.0, which is based on the relative intensities of featured peptides detected in multiple samples; the detection of features was separately performed on each sample and the expectation-maximisation algorithm [74, 75] was used to identify additional overlapping features. Then, an alignment algorithm [76] was employed to align the features of the same peptide from different samples. The contents of extracted proteins in different replicate samples were quantified using a NanoDrop 2000c spectrophotometer (Thermo Scientific, Waltham, USA); for each sample, about 1.5 μg of the protein was analysed via LC-MS/MS. Biological triplicate samples of each stage (i.e. aggregation and alarm) were used in tandem repeats for LC-MS/MS procedure as described above, and the relative concentrations of proteins were compared and presented as the final results. The mass shift between different runs was set to 50 ppm, and 1.0 min was used for evaluating the retention time shift tolerance. Featured peptides with FDR threshold 1%, including PTMs mentioned above, were included in the quantitative analysis. The result of peptides was first filtrated based on: (i) ratio *versus* quality-score (a value indicating the quantifiable level of a peptide) and a fold change of 8 (recommendation of PEAKS Q) was used; (ii) ratio *versus* average-area (MS signal intensity) and set to a fold change of 8; (iii) charge of featured peptides set to between 2 and 5; (iv) fold change of peptide ≥1; and (v) featured peptide detected in more than one sample of the triplicate. Furthermore, protein results were filtered with FDR ≤ 1%, the number of unique peptides ≥1 and fold change ≥1.5. The abundance of neuropeptide precursors were compared, and proteins were clustered using one minus Pearson correlation [77].

### Identification of secreted protein and neuropeptide cleavage sites

Peptide precursors were initially subject to BLASTp and tBLASTn using the corresponding database of NCBI. Protein N-terminal signal sequences were predicted using the SignalP 4.1 [78] and Predisi [79], with the transmembrane domains predicted by TMHMM [80]. For SignalP predictions, positive identifications were made when both neural network and hidden Markov model algorithms gave coincident estimations; D-cutoff values were set to 0.34 (to increase sensitivity) for both SignalP-noTM and TM networks. Herein, a protein was designated as secreted only when it met the criteria of both SignalP and Predisi and did not have a transmembrane domain predicted by TMHMM.

*Biomphalaria glabrata* neuropeptides were predicted by annotating protein sequences derived from the genome and transcriptome to the precursor proteins of known molluscan neuropeptides downloaded from the NCBI website (http://www.ncbi.nlm.nih.gov/). Other identified proteins were defined as novel neuropeptide-like peptides only if they were predicted to: (i) be secretory, and (ii) have multiple cleavage sites based on Neuropred [81]. De novo-only sequenced peptides were subjected to the BLAST against the neuropeptide database, NeuroPep [82], and those with matches were designated as novel neuropeptides of *B. glabrata*. Multiple sequence alignments were created with the Molecular Evolutionary Genetics Analysis (MEGA) software version 6.0 [83]. Sequence presentation and shading of multiple sequence alignments was performed using the LaTEX TEXshade package [84].

## Additional files

Additional file 1: Table S1. Identification and quantification of neuropeptide precursor proteins from non-infected/*S. mansoni*-infected CNS of *B. glabrata*; the identification of genome or transcriptome-derived proteins and protein annotations. (XLSX 197 kb)

Additional file 2: Figure S1. Protein-protein interactions between proteins identified in non-/infected *B. glabrata* CNS and *S. mansoni*. (TIFF 1844 kb)

Additional file 3: Table S2. Proteins on the PPI network and the annotations of relevant proteins of *S. mansoni. (XLSX 173 kb)*

Additional file 4: File S1. List of neuropeptide precursors annotated with signal sequences (predicted by SignalP 4.1, yellow) and cleavage sites (predicted by NeuroPrep, red), supported by ESI-TripleTof MS/MS analysis of *B. glabrata* CNS. Cystine residues are shaded green. (DOCX 19 kb)

Additional file 5: Table S3. Sequences of the precursors of novel neuropeptides, peptides identified by LC-MS/MS and bioactive peptides cleaved from the precursors predicted by Neuropred. (XLSX 19 kb)

Additional file 6: Table S4. Neuropeptide matches identified in the CNS of non-infected (NInf) and *S. mansoni*-infected (Inf) *Biomphalaria glabrata* by LC-MS/MS. Lower case “a” at the C-terminal peptide indicates C-terminal amidation. (XLSX 18 kb)

Additional file 7: Figure S2. Wnt/β-catenin signalling pathway, the recruitment of Axin–GSK3*β* to the membrane is regulated positively by MACF1 and negatively by Bili, whereas Caprin-2 stabilises the LRP6 and Axin–GSK3*β* complex, and MAPKs or GRK5/6 phosphorylates the PPPS/TP motifs of LRP6. *S. mansoni* infection eliminates MACF1 from *B. glabrata*, thereby blocking the signal. (TIFF 1206 kb)

## Abbreviations

CNS: Central nervous system
LAP2: Leucine aminopeptidase 2
LC: Liquid chromatography
MEGA: Molecular evolutionary genetics analysis
MS/MS: Mass spectrometry tandem mass spectrometry
Pep: Pedal peptide
