# Supplementary figures and images for: Changes in the neuropeptide content of Biomphalaria ganglia nervous system following Schistosoma infection
Source: Parasit Vectors. 2017 Jun 2;10:275. doi: 10.1186/s13071-017-2218-1 (PMC5455113; doi:10.1186/s13071-017-2218-1)

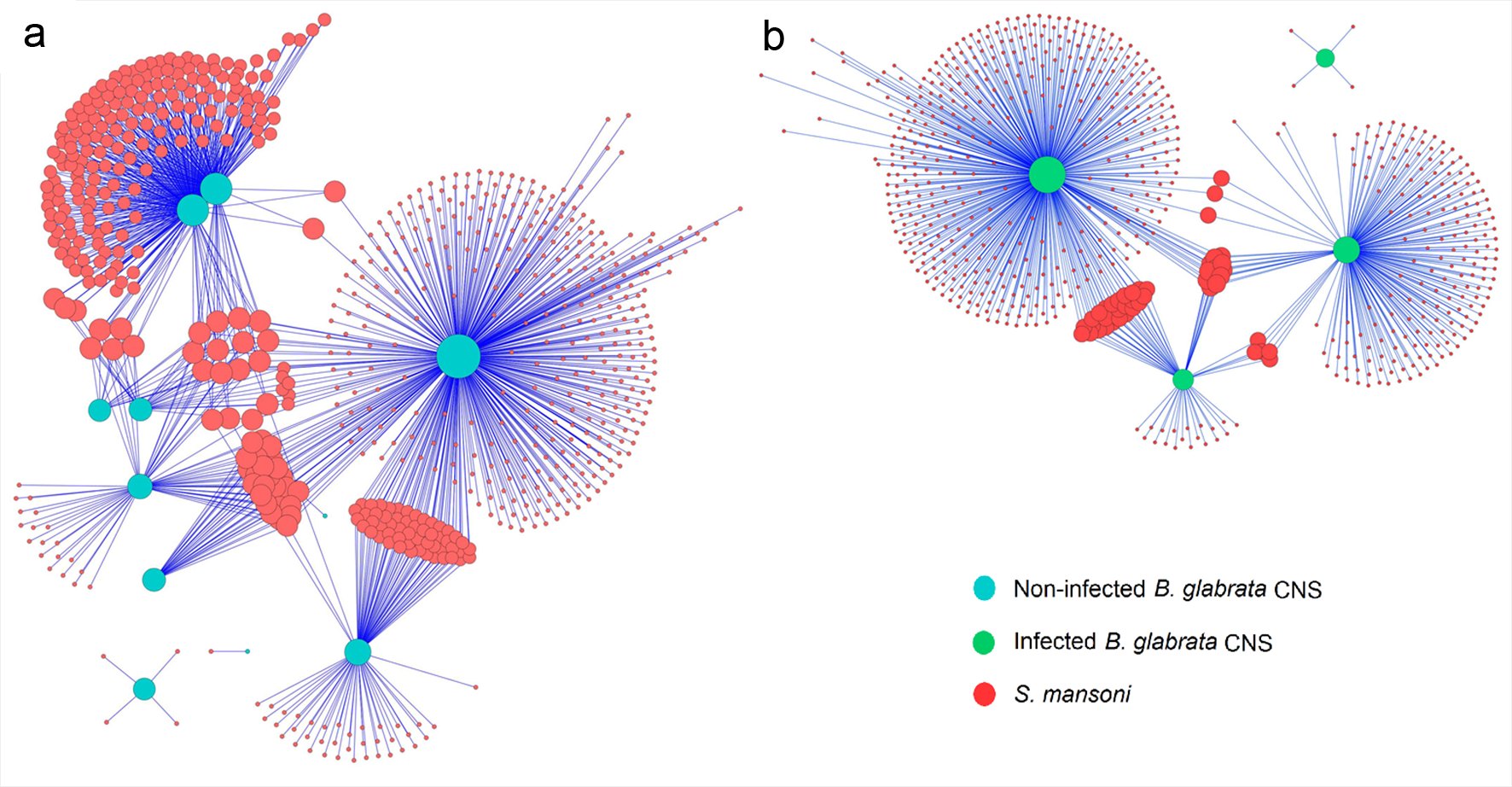

Supplement: Supplementary file 2 — Protein-protein interactions between proteins identified in non-/infected B. glabrata CNS and S. mansoni. (TIFF 1844 kb) [file 13071_2017_2218_MOESM2_ESM.tif]

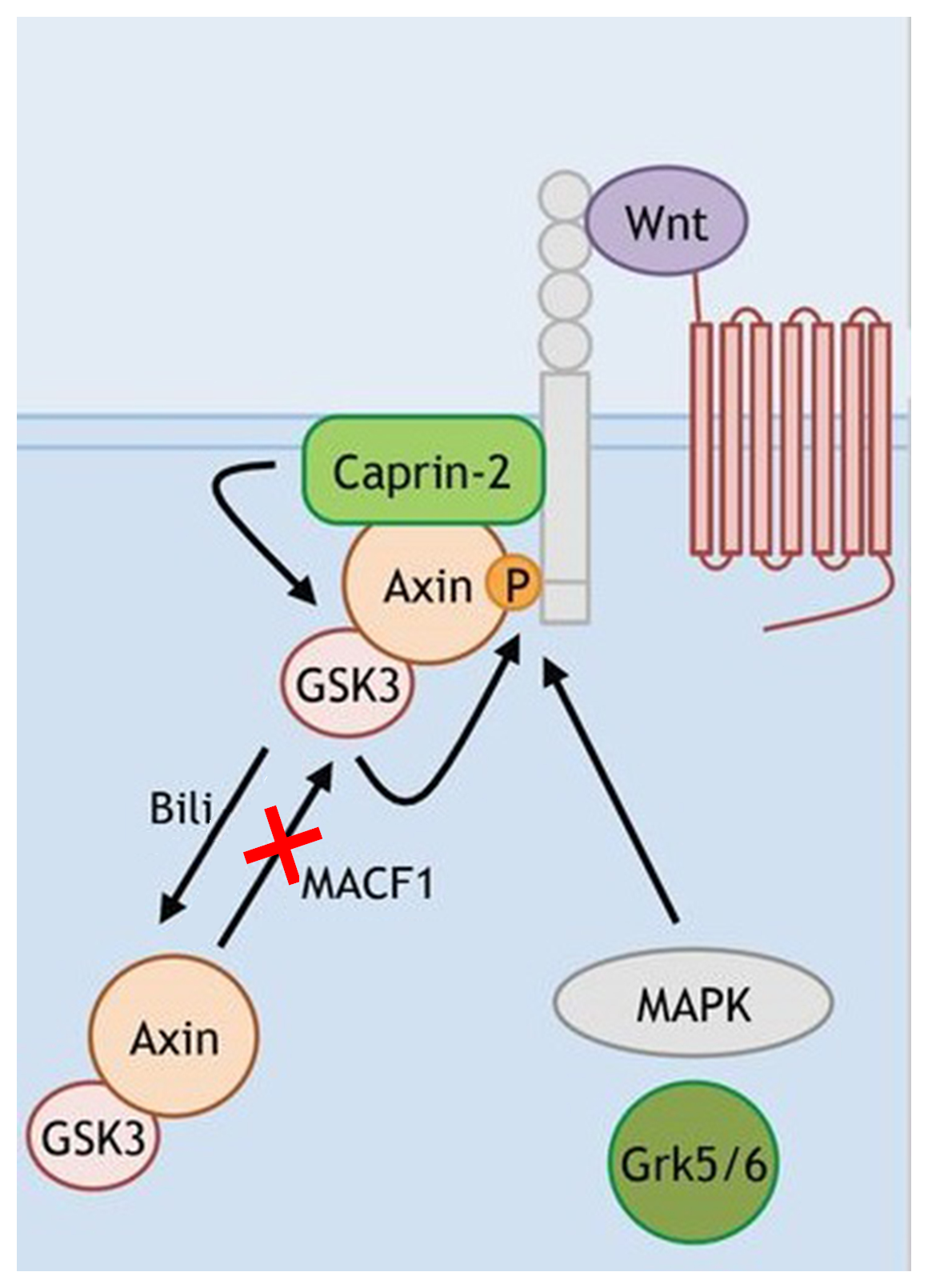

Supplement: Supplementary file 7 — Wnt/β-catenin signalling pathway, the recruitment of Axin–GSK3β to the membrane is regulated positively by MACF1 and negatively by Bili, whereas Caprin-2 stabilises the LRP6 and Axin–GSK3β complex, and MAPKs or GRK5/6 phosphorylates the PPPS/TP motifs of LRP6. S. mansoni infection eliminates MACF1 from B. glabrata, thereby blocking the signal. (TIFF 1206 kb) [file 13071_2017_2218_MOESM7_ESM.tif]
