# Supplementary material for: Changes in the neuropeptide content of Biomphalaria ganglia nervous system following Schistosoma infection
Source: Parasit Vectors. 2017 Jun 2;10:275. doi: 10.1186/s13071-017-2218-1 (PMC5455113; doi:10.1186/s13071-017-2218-1)
Supplement: Supplementary file 4 — List of neuropeptide precursors annotated with signal sequences (predicted by SignalP 4.1, yellow) and cleavage sites (predicted by NeuroPrep, red), supported by ESI-TripleTof MS/MS analysis of B. glabrata CNS. Cystine residues are shaded green. (DOCX 19 kb) [file 13071_2017_2218_MOESM4_ESM.docx]

**Table 1.** List of neuropeptide precursors annotated with signal sequences (predicted by SignalP 4.1, yellow) and cleavage sites (predicted by NeuroPrep, red), supported by ESI-TripleTof MS/MS analysis of *B. glabrata* CNS. Cystine residues are shaded green.

| Achatin | MSTIASYFIMTFVLLGTLHCLAANEVEFLEAAELEPFEDKELLLSDLDRRGFAD**KR**GFAD**KR**GFAD**KR**GFAD**KR**GFAD**KR**GFAD**KR**GFAD**KR**GYGDFREINPLLWKYMRHSQHQPLAIGQNSF**KR**LLAQQGILDN |
| --- | --- |
| APGWamide | MRAHSVSFSSVLLAQIVLLAASLLVELVNSAESSIDTAAQTSSSSESVHTPASRY**KR**APGWG**KR**NGFSEELLEDNENTQELMARLAEY**KR**APGWG**KR**TSSLDIENALSADKKAPGWGKRNAESAFEDDAVDLD**KR**APGWG**KR**APGWG**KR**APGWG**KR**APGWG**KR**APGWG**KR**SVDDYCQTLDQIMDEYIHKALEVKGKKMAECGSTDETNEPLRK |
| AAP12 | MESRVLFTLVSLLLGVSGVLSFSIGRRQERSTDLADALAILQRQRRRVGGGNYVSSESLLDSRPLTLEDVNEWLSKTRSGHGSRDYRYVDPSWVSDGEFKDFDPRLSYNVALDASPLLPQSVKATKQELNDIFNSDDGESNSSKLVHTISDQPEKKKKEEMIELKGDKKIIQKKSVGMDVSEALAVAAERAEEDIDDNLSLDSLTKEEFKTLMKAVGKLQKQAMKLSEKKFDSPDAEKVTVIETVQSEGPEKAVAVVQPASDEELKTVFEETVQPVVKETDIIIEGPQGTKNEKIVEEAVIPVSEPVSNKDIKEAEKELSSAIASQLESELEDVNKEIIQNEEIAKEAEDEILAKNLDALELLNSFS |
| Luqin | MKTSQIILSCCCVLAAVLTICYSKPQWRPQGRFG**KR**TDLSSTTDTD**KR**SPWRPQGRFG**KR**TLADELSVPFFHTREIPVEILFTTKDLENPAFRPVLCTVTAVSGYPVCETALVETRDTDAILDF |
| Buccalin_1 | MLPKNWLHSFVLCLVVLSSCRAYDPDTNEFDGERPEDLVTSDSLDNSEAMD**KR**KLDRCGFHMGIGKRDDEEDGDLEDVYEKRRIDPFAFSGGIGKRRLDRFSFAGGIG**KR**GIDRYGFVAGIG**KR**RLDRFGFSGGIG**KR**GIDRFNFAGGIG**KR**PIDRFSFAGGIG**KR**GFDRYGFYGGIG**KR**PFDRYAFAGGIG**KR**PIDKFGFYGGIG**KR**RLDRFSFAGGIG**KR**IPDLEEVSAAELSEAANDVE**KR**SVPSAKSEKETVKST |
| Buccalin-like | MIVYCLQSHKNMDRALLVCVLKLLLIGVISAAKVQRTSFRWSELPSLRNRNSRVIPSRNTYFNRHE**KR**PFDEIGSGLIG**KR**PFDEIGSGLIG**KR**PFDEIGRSGLIG**KR**PFDEIGSGLIG**KR**PFDEIGSGLIG**KR**PFDEIGSGLIGKRPFDEIGSGLIGKRPFDEIGSGLIGKRPFDEIGSGLIGKRPFNEIGSGLIGKRPFDEIGSGLIGKRPFDEIGSGLIGKRPFDEIGSGLIGKRPFDEIGRSGLIGKRPFDEIGSGLIGKRPFDEIGDGLIGKRPFDEIGSGLIGKRPFDEIGSGLIGKRPFDEIGSGLIGKRNNPGNTQKIFRKYEKIPFIFTDIIA |
| CCAP | MTYEHVSTTLDWLKLVTWLSLTMCTVSVHAEKPGQYLNFPLTSTVNVHSLADDQSPPRLQHLKTSMLDLFSPSPDLESNRALDSAPTTDGGFEKQKRVFCNSFTGCGGKFRGRRRRLKKTFGKRFLNERLAKRPFCNMHGCSNSGKRSGEVLYGDDALIGQLKFSDDELSSLKLNRQLCNSLGGCQNVAPRVGLLERLKQLDSDQTDSLGKRFYSGGFDEDADALAENMGR |
| Cerebrin | MYKNTLSYRTLLALVMVLAITWLTHGTDAISLRPYVGSQYDHKSNQELISLASRMINLLKYGYDFDDGITKRNGGTADTLYNIPDLMHIGRR |
| ELH 2 | MISLVGVAFCLLSSQNSQFVGAMSVHAAASGDRRTNVAVDKEMNKIMASGGGLELMEIPQSPEDETYARAPNSVGESEEDDFPVDDSESVYAHDKSRLVQNNPKLRFNKRYRLRTSKRRLRFQKKKQPYGDDSEYGRPEDSGSAYERDLRGPRLRFHAHDVRKRSAYWGDAAEDLDREQFRQRRSSPFARLVKRVPIGNDLLALADLLFIERQKQAYSALKAMMDEAGKR |
| Enterin | MAAQHCTGFLTFTLLLVTLYSTKADSEQNKMLSLVKPTPGIGKQILRLRFQRRVSPKFGHNFVGKRSFGHNVVWKRSSDFDPTRGADLDHLSIDSRAARFSHSFVGKRAEDLQADEFLRELLERQTIDKLNNNDDGDVNIEERAGPGFPHSFVGKRYDGGESSEDESEDDLSEDKRGAPRFGHSFVGKRNYAVGILNRRGPVFAHNFVGKRNSDDIGKFLDLERRRVGFSHSFVGKRVPPEDFETKPFEQQDVSEHYLEKRSAPIRNKRSLRPSFDHAFIGKRDDDTFDEDIPVDERATPRFEHAFIGKRAPSFSHSFVGKRAPAFSHSFVGKRAPAFSHSFVGKRAPAFSHSFVGKRDEADVAKRAPKYAHAFIGKREAGTEPRLEDNLDDYEEKRASKFSHSFVGKRAPGFSHSFVGKRLPGFSHSFVGKRAPGFIHSFVGKRLPGFSHSFVGKRAPGFSHSFVGKRLPGFSHSFVGKRAPGFSHSFVGKRYPSLNDFNDNFFPLNEDDETEKRSMGFSHSFVGKRLPGFSHSFVGKRDDQDEDLEKRKSFSHSFVGKREDLEDVEKRKMGFSHSFVGKRDALDEDVVKRKMGFSHSFVGKRMGDKHLSNNLEQFSKFESSNSISSNEASSERMADHPIGNMEAQSSASSSKETSSTEESST |
| FCAP_1 | MTIACFALLSLLTVYFTGHADASYIYRSRPLPSDEILRFLSLERDPPADDIGHTSPRFVYRKRSPYSASGLSRFTSLVYEPGSARSYYPLPYQWSRRIGSDFINDDAEKGKRFFDSLGGYEVHSFKKRDIDEGLFSDQDQELYDDKRSIDSLGGFNVHGWKRSIRSPANTRQRYRRDVSCNQGDCDRASSTETTDSSQDADSSKDTDSSQDLEDSLDGFNIRGWKINASQADHVQGSLEKSDLSIIEDKKALDLDVQVDSMF |
| FCAP_2 | NVHGYKRDDDKRALDSLGGFNVHGYKREAETTLVDSTKYSVSHNIHSLQQNDGSDPELAGSSPNVSGSHSSSSDRNLTTKRPTQDKKTHISNVSNNQP |
| FRMFamide | MRTWSHTAILACLTIKWLTIAVAQTVICDDQDLCGVNKRFLRFGRAFDSNADDGFFGYRKQFYRIGRGSFQPYQDKRFLRFGRSDQPDVDDYIRAVLLERQSEEPTYRKRRSPELDEQSDEFAHRMRRSADKAAPAEKDVEKREAETDDAEAEKRFMRFGKRFMRFGRELGYGDKRFIRFGKRFMRFGRDPQDDAEDDTYDADGDFDSDDELAKRFMRFGKRFMRFGKREDAEKRFMRFGKSDEDKRFMRFGKSDSSDDMEKRFMRFGKRESRSVNQESSNSASSGTASHTS |
| FFamide | MKSSLVVLCLTTIIALWSLSECRVDSKDMIQQPLLFGRRGVNPNLNSLFFGKRSSGMEQLVSIREIKHACSVLNSYFDNMESLIGEDESQM |
| FVRIamide | MFINIILISVTVLLHGVSGDIAEDNLEDDKRASSFVRIGRPSSFVRIGRGDNVEDLETDPNYVDLEKKASNFVRIGRYPTMSRFIRIGRTPMEGAGSYEDDSSEEEPIGDDGKRASSFVRIGKRKSSFVRIGKSLAEEDSDEDVDKRASSFVRIGKSPSSFVRIGKAPSSFVRIGKSPSSFVRIGKSPSSFVRIGKVPSSFVRIGRSIENDLKSELDDIDEEKKASSFVRIGKSPNEELVDSEEEKKRASSFVRIGKSGLNDQDLFKRVSSFVRIGKSQGEEDKRVSSFVRIGKSGADEVEDEGKRASSFVRIGKSDTPMDKKASSFVRIGKSSTSPAETSSDSANSAISDEDPINIASRSSAFVRIGKIPSSAFVRIGKNTNLLTAPSENWKLGFRRGSREGQSSFVRIGK |
| Fulicin-like | MLYRRLSPLAVLISSFFLSCVIAVGDTSVKTQTKRTARSPNTLCGSNTADSRTDEDGLVAPLDTKNHYSESSLPRFSGFADNMDSGELLDKQPIVAQEGGTFLNQGNEAFSSDSAESSQKPTAAKRFNEFVGKRSSPTGANVDSDLARELLQRFKSSHKNSDDQEALKQLTLLRSLTADDTSKRQYEFVGKRNYDFLGKRYDFIGKRAPYDFIGKRAQYEFIGKRAYDFLGKRHYDFLGKRSGTNEKGREDEEQDGGKRYSEFLGRRKRTEEQGSALMTDSARLAALLQNNSLRKRISEMLMKQRLAEQVPEFVGK |
| Insulin-like_peptide_1 | MAYVSRLLLLLTLAHSTSVTVAEYDHTCNVLSRPHRNGRCGRLLVDTVNLLCNSFSGLLVKRDTTERVNENLKHILLNKKEALSYLTKRETRGSIVCECCYHTCTISELLKYCSYDVYNSKHAAAYRSSHQGQ |
| Insulin-like_peptide_3 | MLLTTAMLVLVLKVSGVHGVATKVCTSTDRSNSRGLCTGHLYSTIMLACDIFRPVNKRDVMTDPVNTMDDIVLAKPNALSFLTKKQSSFTVICECCFNQCTVSEMLDYCSLFGRGKKI |
| LFRFa | MDHSTLALALTFFTAIVCHVYSEEMNHVSALNTLEDHQETHQPQKRSPASSPALYSEDLEADSGLEEPMDDMDKKNTLFRFGKRQGAWFRYGKRAGTLLRFGKRGTLLRFGKRGGSLLRFGRGGNSDADFSEDDKRTLFRFGKRSDLEEIVRDALAREELSNSPWYMENEPVKRGVNGFHWGQESEN |
| Myomodulin 1 | MFINIILISVTVLLHGVSGDIAEDNLEDDKRASSFVRIGRPSSFVRIGRGDNVEDLETDPNYVDLEKKASNFVRIGRYPTMSRFIRIGRTPMEGAGSYEDDSSEEEPIGDDGKRASSFVRIGKRKSSFVRIGKSLAEEDSDEDVDKRASSFVRIGKSPSSFVRIGKAPSSFVRIGKSPSSFVRIGKSPSSFVRIGKVPSSFVRIGRSIENDLKSELDDIDEEKKASSFVRIGKSPNEELVDSEEEKKRASSFVRIGKSGLNDQDLFKRVSSFVRIGKSQGEEDKRVSSFVRIGKSGADEVEDEGKRASSFVRIGKSDTPMDKKASSFVRIGKSSTSPAETSSDSANSAISDEDPINIASRSSAFVRIGKIPSSAFVRIGKNTNLLTAPSENWKLGFRRGSREGQSSFVRIGK |
| Myomodulin 2 | MKGTFFLSISVAVTCLLSLGQSEEAKVQSVHSDQTNDAADHPRLKRGLAMLRLGKRDSADIDNLAELLYQMRESEELGAGSEGEDPEEVEELEVPAQHPRVRRSAQSPTENPHHEVLENSSNVEEFVEHEPRLVDDSYFYVFPDGSEELEPSEGGEDADNFDELEKRSMKMLRLGRSATDDDIFTNDKRQLKMLRLGKRGDASFDEEFDDEMDKRSLKMLRLGKRPMNMLRLGKRPMNMLRLGKRPMNMLRLGKRPMNMLRLGKRPADLTDAAEDEKRSMKMLRLGKRSAH |
| NdWF | MLSTLARITLTVLLVAVVVLPSHGNFYGKRGDKDDLYSAILQAAESPSRRDLTADFDTESAIESVLRMARQQESKFTETKRG |
| NKY | MASSFLAYNVILFAVCLSFAVATFDIEEDVNDYPDQEKRTYATTDATLETILNVLKSHAQSLRQLESTVYEQKRSGFRSRSGDDLLGGVKRRMVWQPLGYLPASIRVQHGSQGPQRPEMQDTGSSVFRYG |
| NPY | MQKILFVSLLVLSLAVMEIVCMDNMLTPPERPAEFKNANELRKYLKALNEYYAIVGRPRFGKRNGASILNDLFRQNGDEFADYGANWADI |
| Pedal Peptide 1 | MKMLSTVTIYSAIVLFAAVVSIQADEELSSNTNHNEIKRSVGTENEVDLDKRPFDSISGSHGLSGFAKRPFDSISGSHGLSGFAKRPFDSISGSHGLSGFAKRPFDSISGSHGLSGFAKRPFDSISGSHGLSGFAKRPFDSISGSHGLSGFAKRSMDEESLYGAYKESDDDIYRREIEEEDDKLFNVAEEELQTVSENTV |
| Pedal peptide 2 | MRTELFLLVILPYSLMSAEVPDGDIKKKSQSQVFQYETRPGDPQGSEMNKRSIDSIGSGFIKRPLDSIGSSFIKKGIDSIGSAFIKRPIDSIGSSFVKKNIDSIGSAFIKRPLDSIGSSFIKRRFHHYAFNKRPLDSIGSSFIKRGIDSIGSGFIKRPLDSIGSSFIKRGIDSIGSGFIKRPLDSIGSSFIKRGIDSIGSGFIKRPLDSIGSSFIKRGIDSIGSGFIKRPLDSIGSSFIKRGIDSIGSGFIKRPLDSIGSSFIKRGIDSIGSGFIKRPIDSIGSSFIKRGIDSIGSGFIKRPLDSIGSSFIKRGIDSIGSGFIKRPIDSIGSSFIKKSIDSIGSGFIKRPIDSIGSSFVKKGIDSIGSGFIKRPLDSIGSSFIKKSLNSIGSGFIKRPIDSIGSSFIKRDYDEEMEDDKRALDSIGSSFIKRPDDDVEEDFENGQLVRGKRSPTDVVVNEPSDSSYDAMAAGLHRNDMVHAQLPLWFAEMEIPEYLLEEAQHNQQLRR |
| Pedal peptide 3 | MPCFIIFFGVCHYVLMSLHNLSTDDYLDSKESSQENVDKRPFDRIGTSSFTSFGKRPFDRIGTSSFTSFGKRPFDRIGTSAFTSFGKRPFDRIGNSAFASFGKRPFDRIGNSAFASFGKRPFDRIGNSAFTSFGKRPFDRIGNSAFTSFGKRPFDRIGNSAFTTFGKRPFDRIGTSVFTSFGKRPFDRIGNSAFTTFGKRPFDRIGSSAFTSFGKRPFDRIGTSAITSFGKRPFDRIGNSAFTTFGKRPFDRIGSSAFTSFGKRPFDRIGTSSFTSFGKREDEEGAENFEVTSVNHLEDAGNAKMKEASNKDNSSLRKKRALESNSKTERKEAAIVKREAEKRTAEGELTLSMVANNADLSGPISKRFSGTFNRFDNEGDDNSNEISSDKLDEFLNKRRFDSISDSSAFNHFGKRRFDRISKNSQFNPFGKRRLDRISSISGFGKFGKRKFDSIADGSRFSSFGKRRFDRISSGFSHFGKRRFDRIDRGSAFSRFGKRNYPFNLGKSSFDRIDKNSAFNAFGKRDWEPVMIDENMLEPTYSQDLDLVPENSRSFSKPFKVLIDSPWPEQHINNAIEGLGYHDDEAEKGLTDMDESSDEEAKQLVKYLLNL |
| Pedal peptide 4 | MPSSTFPISSETMALLLLLTVPSLLLTSVQGVPAQVSKDVKLPASAAVTQSYVGISPSSTAKAEINNSLSVSSIVTKDKPAADPKSSKPGLHADVVKRSALQEVVAPSGLGHRHKRQFNDEDRSGDLSREELGENSESNDAKATFELMLNGVSGSGEKYDAKRHFDSIGESGLSGIHQNYMGRRNNIDISSLLRNRLSKRQFDSIGESSLSGIHQNFIGRRNFDSIGESSLSGLYQNHMGKRQFDLISESELTGIHQNYLEPRRNNRNNRWQYSPKRNFDLVGRGGMSGMYKNFIGKRDGRFFDGKRRFDSIGESGLSGIHQNYLGKRSFDSIGASGLSGMNQNFYGKRSFDSIGSSGLSGIHQNYLGKRSFDSIGESGLSGINQNFYGKRNFDS |
| Pleurin | MASITHMEKMVILLSMFCWTSYAVFYYKHEPDYPRLGKRTSYSDDKLQGEGSNPQLMLPDPAFTNLNRRGIFTQGSAGVPRLGRRNLDTDKRGVFTQANGGYIRMGKRDDPWNDKRGVFTQGQIGSPRIGRQGASDIWSDGVNQVLQKMAEFKEDNDEEKAGQGQMDVGNDVLLQPTSIPYLFIEMDTDNDGKLSKEEFVSGIQILKSQTSYC |
| Prohormone-1 | MNSSLLTLATVLFAAALLLSSSVGGSPAELIQKEVKRSIQHQPQSAHMEIADRLLRRILDVTYETLQDLGASNEDLERVQQKRNYIQTCYFQAISCY |
| PTSP-like | MTLALTLCHVILICLLQDTALADRTLQLNEAVEDPSSTSASAENVGDQPSEKRHWSQFRSWGKRSLDDDALSKRWKEMSVWGKREADLLSNHQDMDYLYPAIVWEGSQMSGANPELDKKWKEMSVWGKRNVDPELEKRWKQMAVWGKRSSDPELEKKWKEMAVWGKRDFDPELEKKWKEMAVWGKRDNDPELEKKWKEMAVWGKRDFYPELDKKWKEMSVWGKRDFDPELEKKWKEMSVWGKRDFDPELEKKWKEMSVWGKRDFNPELEKKWKEMSVWGKRDFNPELEKKWKEMSVWGKRDFDPELEKKWKQMSVWGKRGVDNNGKQTIRHTRKWRWANLGAKRPSWSSTGFSSWGKRSEDLDLHDVKEYLQKTLPVSELEETPQEAGELKSQDQGTPLSKT |
| PRQFVamide | MLPQVSTFLTLLAILHLPKSCHSENVSSDPGSGDALQSLDDLRWQSFSSDGNSDVDKRVREFVGKRSEDQFYNENDAIEKRPRQFVGKRSEDKRVREFVGKRVREFVGKRDNGDIDTDKRVREFVGKRNSYGDEFDNADDVDRFLAEKRPRQFVGKRSGPYFYDEIISNEKRPRQFVGKRSSPYFYDEILNNEKRPRQFVGKRDNLEDIFLNEEKRPRQFVGKRYIADEDFLIDAEKRPRQFVGKRYSFALDNLVEKRPRQLVGKRNFDEVLTDAAQRNAITNYILHRLVEESPELMDGVLTEDGQLRTKRSVSDCEDDLKKSLTELFSKDDEDLRDRFSLDKRIREFVGKRSPSSCVDLVKRVREFVGKRAEKRPRQFQGKRSTDSQTKGSR |
| sCAP_1 | MELTLLQATVTLAVLVLVGGEKQTQKPYIAFPRMGRSEIQKKPQLPLNFIVPRKRAMPKGFIIPRKRAEDDNGYLAFPRMGRSQLKPETFFDNGACCGVGVKAEYFVSQDGKEVIRSSCAPHLVCCEGLREINDEKTDGVYFSLCIPDTPLAEESGVRSSEILSSLKRLLEK |
| sCAP_1 | MELTLLQATVTLAVLVLVGGEKQTQKPYIAFPRMGRSEIQKKPQLPLNFIVPRKRAMPKGFIIPRKRAEDDNGYLAFPRMGRSQLKPETFFDNGACCGVGVKAEYFVSQDGKEVIRSSCAPHLVCCEGLREINDEKTDGVYFSLCIPDTPLAEESGVRSSEILSSLKRLLEK |
| Whitnin | MEFPTFPFFVLVLTIVSVSLSNSLPTRTEDVLQEANMALEKRPKYMDTRRELDMFKDLVLMSLQELVDEERVNPTVLLEDESSKTVEKRERYMGICMKKQYNNFVPVPCLRSGR |
